# Complications of Elective Lumbar Spine Fusion in Patients With COVID-19

**DOI:** 10.7759/cureus.104412

**Published:** 2026-02-27

**Authors:** Henry Avetisian, William J Karakash, Daniel Rusu, Aidan Lindgren, Chimere O Ezuma, Jeffrey C Wang, Raymond J Hah, Ram K Alluri

**Affiliations:** 1 Department of Orthopedic Surgery, University at Buffalo Jacobs School of Medicine and Biomedical Sciences, Buffalo, USA; 2 Department of Orthopedic Surgery, Keck School of Medicine of USC (University of Southern California), Los Angeles, USA; 3 Department of Orthopedics, Keck School of Medicine of USC (University of Southern California), Los Angeles, USA

**Keywords:** covid-19, healthcare costs, lumbar fusion, perioperative complications, spine surgery

## Abstract

Introduction: The COVID-19 pandemic led to widespread delays in elective surgery, and prior studies have shown increased perioperative risk when surgery is performed soon after infection. While recommendations exist for delaying elective arthroplasty following COVID-19, data guiding the timing of elective spine surgery remain limited. This study evaluates complication rates in patients undergoing elective lumbar fusion within 14 days of a COVID-19 diagnosis using a large national database.

Methods: The PearlDiver Mariner Database was queried to identify all adult patients who underwent primary elective lumbar fusion between 2020 and April 2023. Patients diagnosed with COVID-19 within 14 days preoperatively were included, while those with COVID-19 diagnoses 15-90 days before surgery were excluded. A control cohort without COVID-19 diagnoses within 90 days of surgery was established. Demographics, length of stay, 30-day costs, readmissions, and postoperative complications were analyzed. Nearest-neighbor 10:1 propensity score matching was performed based on age, Elixhauser Comorbidity Index (ECI), and gender. Multivariable logistic regression adjusted for age, ECI, gender, and number of levels fused, assessed the association between recent COVID-19 infection and postoperative outcomes with reported odds ratios (OR).

Results: Of 128,337 patients undergoing lumbar fusion, 548 (0.43%) had a COVID-19 diagnosis within 14 days of surgery. After propensity matching, patients with recent COVID-19 infection had significantly longer hospital stays (7.96 vs. 4.46 days, p<0.001) and higher 30-day costs ($9,464 vs. $7,543, p<0.001). Recent COVID-19 diagnosis was independently associated with increased risk of 30-day (OR 14.54, p<0.001) and 90-day (OR 11.67, p<0.001) all-cause readmissions, intraoperative transfusion (OR 2.81, p<0.01), and multiple medical complications, including pneumonia (OR 6.87), respiratory failure (OR 3.86), acute kidney injury (AKI, OR 2.41), sepsis (OR 1.86), shock OR 3.12), and cardiac arrhythmias (OR 1.78) (all p<0.05). No significant differences were observed in surgical complications or incision and drainage (I&D) rates.

Conclusion: Patients undergoing elective lumbar spine fusion within 14 days of a COVID-19 diagnosis experience significantly higher rates of medical complications, hospital readmissions, length of stay, and healthcare costs. These support consideration of delaying non-urgent elective lumbar fusion for at least two weeks following COVID-19 infection, with careful assessment of patient comorbidities and infection severity.

## Introduction

The COVID-19 pandemic significantly disrupted healthcare systems worldwide, leading to the widespread cancellation of elective surgeries across the United States to preserve healthcare resources and reduce transmission risks. Although no longer classified as a national emergency, COVID-19 continues to affect millions globally currently [1]. Several studies have demonstrated that patients with COVID-19 who undergo elective orthopedic surgeries are at increased risk of severe complications, including venous thromboembolism (VTE), acute myocardial infarction (AMI), pneumonia, respiratory failure, intensive care unit admissions, and death [2,3]. Previous studies have recommended delaying elective total joint arthroplasty for at least eight weeks after a COVID-19 infection, with a minimum postponement of 12 weeks for patients who experienced severe or critical illness [4]. This delay has been linked to lower complication rates and mortality compared to performing surgery while the patient has an active COVID-19 infection [4]. Spinal fusion surgeries differ procedurally from total joint arthroplasty and require an inpatient hospital stay, thereby adding additional hospital costs.

However, limited data exist regarding outcomes for patients undergoing elective spine surgery following a recent COVID-19 diagnosis. Notably, there are no formal guidelines from spine surgery professional societies, such as the North American Spine Society (NASS), to dictate the optimal timing for proceeding with surgery in these patients; however, the American Society of Anesthesiologists (ASA) and Anesthesia Patient Safety Foundation (APSF) advise against elective surgeries within two weeks of a COVID-19 infection. A study by Chan et al. identified elevated rates of VTE, sepsis, and both 30- and 90-day mortality among patients who tested positive for COVID-19 within two weeks of elective lumbar fusion, leading to a recommendation of delaying surgery for at least two weeks in non-urgent cases [5]. However, this recommendation sparked debate due to weaknesses in the study’s design and its reliance on a single database [6]. 

A 2021 survey of AO Spine members revealed that many spine surgeons had canceled up to 25% of their cases due to COVID-19-related delays [7]. Thus, spine surgeons are confronted with the dilemma of whether to proceed with surgery in patients recently diagnosed with COVID-19 or to delay the operation, with each option posing potential risks and benefits. 

This study aims to address this clinical dilemma by analyzing rates of 30-day postoperative (medical and surgical) complications, 30-day and 90-day all-cause readmission rates, and 30-day and 180-day incision and drainage (I&D) in patients undergoing elective lumbar fusion surgery within 14 days of COVID-19 diagnosis using a large national database. We seek to evaluate the validity of the recommended two-week delay period by leveraging a different national database. We hypothesize that patients with a recent COVID-19 diagnosis will experience significantly higher rates of complications following elective lumbar fusion.

## Materials and methods

Data source and study population

The PearlDiver Mariner Database (PearlDiver Technologies, Fort Wayne, IN, USA), a national database that contains 170 million de-identified patients, was queried for all patients who underwent lumbar fusion between 2020 and April 2023. PearlDiver contains patient demographics, diagnostic, and procedural data. Since this study used anonymized data from PearlDiver and was retrospective in nature, informed consent was waived, and Institutional Review Board (IRB) approval was not required. To protect patient privacy, any values less than 11 are withheld, by default, in the PearlDiver database.

Identification of study cohorts

Patients who underwent primary lumbar fusion surgery were identified using Current Procedural Terminology (CPT) codes, including anterior, lateral, and posterior approaches (Table 1). Exclusion criteria included patients younger than 18 years, those with rheumatoid arthritis, cauda equina syndrome, >5 spinal levels fused, previous lumbar fusion, less than 90 days of follow-up before or after surgery, or surgical indications related to trauma, malignancy, or infection. 

**Table 1:** Current Procedural Terminology (CPT) and International Classification of Disease 10th (ICD-10) edition codes used for patient inclusion and stratification

Definition	Code
COVID-19	U07.1
Lumbar fusion	CPT-22633, CPT-22612, CPT-22634, CPT-22558, CPT-22614, CPT-22585, CPT-22630, CPT-22632

**Figure 1:**
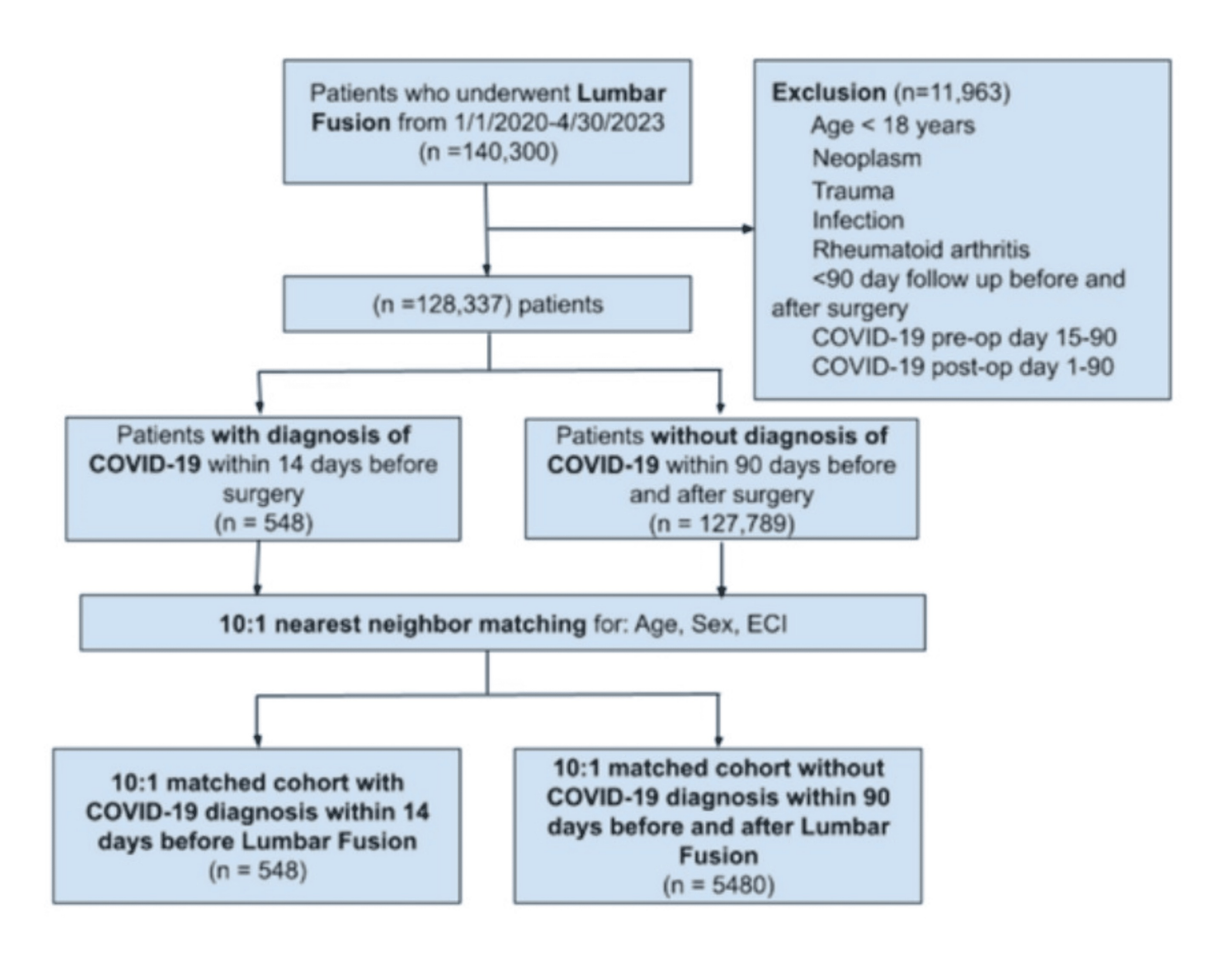
Patient selection methodology ECI = Elixhauser Comorbidity Index

The International Classification of Diseases 10th (ICD-10) edition code, U07.1, was used to identify patients with a diagnosis of COVID-19 [8]. Patients diagnosed with COVID-19 within 14 days before their procedure were included in the study. A two-week preoperative COVID diagnosis was selected based on prior literature demonstrating significantly increased postoperative complication rates, without evidence of elevated risk beyond two weeks [5]. In addition, to ensure first-time diagnoses and avoid diagnosis carryover from prior encounters, patients with a COVID-19 diagnosis between 15- and 90-days pre-procedure were excluded. This was implemented to address potential overrepresentation of preoperative COVID-19 diagnosis, but may limit generalizability. The control group comprised patients without a COVID-19 diagnosis within 90 days before or after surgery. Figure 1 provides an overview of this methodology.

Outcomes

This study aimed to characterize the prevalence of COVID-19 among patients undergoing elective lumbar fusion within two weeks of surgery and evaluate the associated risks. Primary outcomes included hospital length of stay, total 30-day cost, intraoperative transfusion, 30-day postoperative complications, 30-day and 90-day all-cause readmission rates, and 30-day and 180-day I&D rates. All-cause readmission rates were captured by a new instance of an inpatient record. The total 30-day cost is defined as payer reimbursement. Surgical complications included dehiscence, hematoma, spinal cord injury (SCI), surgical site infection (SSI), and hardware/graft complications. Medical adverse events included acute kidney injury (AKI), acute myocardial infarction (AMI), blood loss anemia, cardiac arrhythmias, deep vein thrombosis (DVT), pneumonia, pulmonary embolism (PE), respiratory failure, renal failure, sepsis, shock, altered mental status (AMS), gastrointestinal/genitourinary (GI/GU) adverse events, bacterial infection, and transfusion. 

Statistical analysis 

The Student’s t-test and Pearson chi-squared test of independence assessed differences in patient characteristics, smoking status, and rates of comorbidities between lumbar fusion patients with and without a preoperative COVID-19 diagnosis. Nearest-neighbor 10:1 propensity score matching was then used to create matched cohorts based on age, ECI, and gender. A 10:1 propensity score match with a caliper of 0.2 was performed to maximize precision while preserving covariate balance. A many-to-one match was feasible because a large pool of controls was available and baseline covariate distributions showed adequate overlap. Matching was deemed appropriate as standardized mean differences (SMD) <0.1 for all variables (Table 2). Length of stay and 30-day total reimbursement were compared with the Student’s t-test. 

**Table 2:** Standardized mean differences following 10:1 propensity score matching SMD = standardized mean difference; ECI = Elixhauser Comorbidity Index

Variable	Before matching SMD	After matching SMD
Age	-0.199	-0.017
ECI	0.412	-0.010
Female	-0.017	0.006
Male	0.017	-0.006

A multivariable logistic regression analysis was performed to assess the impact of a preoperative COVID-19 diagnosis on complications, readmissions, hardware removal, and I&D rates. The number of spinal levels fused, age, ECI, and gender were included as covariates in the regression model. All statistical analyses were carried out using RStudio (Version 4.4.2, Posit Software, Boston, MA) within the PearlDiver Mariner Database platform. A p-value of less than 0.05 was considered statistically significant.

## Results

Demographics

Demographic data is summarized in Table 3. Between 2020 and April 2023, we identified 128,337 patients who underwent lumbar fusion, of whom 548 (0.43%) had a COVID-19 diagnosis within 14 days of the procedure. Patients in the COVID-19 cohort were younger on average (59.79 ± 12.47 years vs. 62.44 ± 12.30 years, t=4.958, p<0.001) and had a higher number of comorbidities (ECI = 7.60 ± 4.20 vs. 5.89 ± 3.75, t=-9.513, p<0.001). Additionally, they had higher rates of diabetes (48.0% vs. 36.95%, *X*^2^=28.370, p<0.001) and history of tobacco use (57.85% vs. 49.31%, 16.050, p<0.001). Propensity matching based on age, ECI, and gender resulted in 5,480 patients in the control group and 548 patients in the COVID-19 group.

**Table 3:** Lumbar fusion patient demographics stratified by preoperative COVID-19 diagnosis ECI = Elixhauser Comorbidity Index; p-value <0.05 is significant.

Variable	Control	COVID-19	Test statistic (t-value or X^2^)	p-value
Unmatched cohorts				
n	127,789	548		
Age	62.44 ± 12.30	59.79 ± 12.47	4.958	<0.001
ECI	5.89 ± 3.75	7.6 ±4.20	-9.513	<0.001
Male	55,306 (43.3%)	240 (43.8%)	0.063	0.841
Female	72,483 (56.7%)	308 (56.20%)	0.063	0.841
Diabetes	47,222 (37.0%)	263 (48.0%)	28.370	<0.001
Tobacco use	63,013 (49.3%)	317 (57.9%)	16.050	<0.001
10:1 Propensity matched cohorts				
n	5,480	548		
Age	59.9 ± 12.43	59.79 ± 12.47	0.197	0.993
ECI	7.61 ± 4.20	7.61 ± 4.23	0	0.999
Male	2402 (43.8%)	240 (43.8%)	0	1
Female	3078 (56.2%)	308 (56.2%)	0	1
Diabetes	2547 (46.5%)	263 (48.0%)	0.423	0.527
Tobacco use	3022 (55.2%)	317 (57.9%)	1.366	0.243

Length of stay and 30-day total cost

Patients in the COVID-19 cohort had a significantly longer hospital length of stay (7.96 ± 10.57 days vs. 4.46 ± 4.78 days, t=-8.973, p<0.001). Furthermore, the total 30-day cost was significantly higher for the COVID-19 cohort ($9,464 ± 10,433 vs. $7,543 ± 9,736, t=-4.412, p<0.001) (Table 4).

**Table 4:** Patient hospital length of stay and total cost following lumbar fusion p-value <0.05 is significant. Cost data reported in USD.

Variable	Control	COVID-19	t-value	p-value
Length of stay	4.46 ± 4.78	7.96 ± 10.57	-8.973	<0.001
30-day total cost	$7,543 ± 9,736	$9,464 ± 10,433	-4.412	<0.001

All-cause readmission, intraoperative, and postoperative complications

Multivariable logistic regression revealed that patients with a COVID-19 diagnosis within 14 days of lumbar fusion had significantly higher risks for all-cause hospital readmissions at 30-day (52.9% vs. 7.5%, OR=14.54 (11.91-17.78), Wald z=14.743, p<0.001) and 90-day (52.9% vs. 9.1%, OR=11.67 (9.61-14.19), Wald z=13.473, p<0.001) intervals. However, patients with preoperative COVID-19 did not have increased risk of I&D at 30-day (4.0% vs. 5.1%, OR=0.79 (0.49-1.20), Wald z=-1.055, p=0.29) and 180-day (6.9% vs. 6.9%, OR=1.0 (0.70-1.40), Wald z=0, p=0.99) intervals following surgery. Intraoperatively, they faced a higher risk of blood transfusions (OR=2.81 (1.39-5.70), Wald z=2.850, p<0.01) (Table 5).

**Table 5:** Patient transfusions, all-cause readmissions, and incision and drainage procedures following lumbar fusion *Indicates reported population of less than 11. For patient privacy, values of less than 11 are withheld in the PearlDiver database. OR = odds ratio, 95% CI = 95% confidence interval, p-value <0.05 is significant.

Variable	Control	COVID-19	OR	(95% CI)	Wald z-statistic	p-value
Same-day transfusion	36 (0.7%)	*	2.81	(1.39-5.70)	2.850	<0.01
All-cause readmissions						
30 days	413 (7.5%)	290 (52.9%)	14.54	(11.91-17.78)	14.743	<0.001
90 days	499 (9.1%)	290 (52.9%)	11.67	(9.61-14.19)	13.473	<0.001
Incision & drainage						
30 days	277 (5.1%)	22 (4.0%)	0.79	(0.49-1.2)	-1.055	0.294
180 days	379 (6.9%)	38 (6.9%)	1	(0.7-1.4)	0	0.994

Regarding postoperative medical complications, the COVID-19 group faced increased risk of pneumonia (5.3% vs. 0.7%, OR=6.87 (4.20-11.08), Wald z=7.196, p<0.001), shock (OR=3.12 (1.30-6.73), Wald z=2.592, p<0.01), respiratory failure (9.5% vs. 2.6%, OR=3.86 (2.75-5.35), Wald z=7.038, p<0.001), AKI (7.5% vs. 3.2%, OR=2.41 (1.67-3.40), Wald z=4.459, p<0.001), AMS (4.0% vs. 2.0%, OR=2.03 (1.24-3.19), Wald z=2.643, p<0.01), sepsis (2.7% vs. 1.4%, OR=1.86 (1.02-3.17), Wald z=2.035, p<0.05), cardiac arrhythmias (16.4% vs. 9.8%, OR=1.78 (1.39-2.27), Wald z=4.493, p<0.001), and all 30-day medical complications (39.6% vs. 24.0%, OR=2.05 (1.71-2.46), Wald z=6.468, p<0.001) (Table 6).

**Table 6:** Thirty-day medical complications following lumbar fusion *Indicates reported population of less than 11. For patient privacy, values of less than 11 are withheld in the PearlDiver database. OR = odds ratio, 95% CI = 95% confidence interval, p-value <0.05 is significant. AKI = acute kidney injury, AMI = acute myocardial infarction, DVT = deep vein thrombosis, PE = pulmonary embolism, AMS = altered mental status, GI = gastrointestinal, GU = genitourinary

Variable	Control	COVID-19	OR	(95% CI)	Wald z-statistic	p-value
AKI	177 (3.2%)	41 (7.5%)	2.41	(1.67-3.4)	4.459	<0.001
AMI	22 (0.4%)	*	2.21	(0.74-5.42)	1.589	0.112
Blood Loss Anemia	52 (1.0%)	*	1.13	(0.43-2.44)	0.279	0.78
Cardiac Arrhythmias	539 (9.8%)	90 (16.4%)	1.78	(1.39-2.27)	4.493	<0.001
DVT	41 (0.8%)	*	1.94	(0.84-3.94)	1.695	0.09
Pneumonia	39 (0.7%)	29 (5.3%)	6.87	(4.2-11.08)	7.196	<0.001
PE	*	*	6.1	(1.25-25)	2.040	<0.05
Renal Failure	405 (7.4%)	48 (8.8%)	1.19	(0.86-1.61)	1.074	0.283
Respiratory Failure	142 (2.6%)	52 (9.5%)	3.86	(2.75-5.35)	7.038	<0.001
Sepsis	79 (1.4%)	15 (2.7%)	1.86	(1.02-3.17)	2.035	<0.05
Shock	24 (0.4%)	*	3.12	(1.3-6.73)	2.592	<0.01
AMS	107 (2.0%)	22 (4.0%)	2.03	(1.24-3.19)	2.643	<0.01
GI/GU	37 (0.7%)	*	2.11	(0.91-4.33)	1.894	0.058
Transfusion	92 (1.7%)	14 (2.6%)	1.47	(0.8-2.52)	1.325	0.185
Bacterial Infection	121 (2.2%)	33 (6.0%)	2.16	(1.4-3.21)	3.563	<0.001
All Medical	1313 (24.0%)	217 (39.6%)	2.05	(1.71-2.46)	6.468	<0.001

No significant differences were found regarding 30-day surgical complications (all p>0.05, Table 7).

**Table 7:** 30-day surgical complications following lumbar fusion *Indicates reported population of less than 11. For patient privacy, values of less than 11 are withheld in the PearlDiver database. OR = odds ratio, 95% CI = 95% confidence interval, p-value <0.05 is significant. SCI = spinal cord injury, SSI = surgical site infection

Variable	Control	COVID-19	OR	(95% CI)	Wald z-statistic	p-value
Dehiscence	68 (1.2%)	11 (2.0%)	1.58	(0.79-2.89)	1.393	0.164
Hematoma	21 (0.4%)	*	1.02	(0.16-3.51)	0.023	0.982
SCI	35 (0.6%)	*	1.39	(0.48-3.27)	0.694	0.49
SSI	117 (2.1%)	14 (2.6%)	1.17	(0.64-1.99)	0.558	0.577
Hardware	178 (3.3%)	16 (2.9%)	0.89	(0.51-1.45)	-0.436	0.663
All Surgical	403 (7.4%)	53 (9.7%)	1.32	(0.97-1.78)	1.820	0.069

## Discussion

COVID-19 has been shown to increase the risk of postoperative complications across multiple surgical specialties [9,10]. This study aimed to further elucidate its impact on patients undergoing elective lumbar fusion with a COVID-19 diagnosis within 14 days of surgery. Our findings revealed that these patients had longer hospital length of stay and increased risks for 30-day and 90-day hospital readmissions, intraoperative blood transfusions, and medical complications. These elevated risks align with previous findings, such as those by Chan et al., who reported increased risks for VTE (OR=2.29), sepsis (OR=1.56), AKI (OR=1.3), and higher 30-day (OR=5.55) and 1-year mortality (OR=2.7), potentially attributing to the significantly greater readmission rate in the COVID-19 cohort [5].

Notably, our study highlighted an increased risk for AKI, a known complication of COVID-19, that affects up to 9% of infected patients and nearly 46% of those hospitalized. Concomitant COVID-19 infection and severe AKI can result in the need for emergent dialysis in 19% of these patients, and in many cases, patients do not return to baseline kidney function at discharge [11,12]. This risk may be further exacerbated by factors associated with lumbar spine surgery, such as intraoperative trauma, hypovolemia, and anesthesia-induced vasodilation and decreased cardiac output [13].

We also observed an increased risk of sepsis in patients with a recent COVID-19 diagnosis. COVID-19 is known to cause a dysregulated immune response that can predispose patients to secondary bacterial and fungal infections. Additionally, the virus damages lung tissue and surrounding infrastructure, facilitating bacterial invasion and adherence [14]. The proinflammatory state induced by COVID-19 further accelerates the progression of sepsis to multi-organ dysfunction and septic shock [15]. These findings are consistent with our observed increases in AKI, respiratory failure, altered mental status, and shock. Deng et al. reported that patients with a COVID-19 diagnosis within four weeks of elective surgery had higher risks of respiratory failure (OR=3.36) and sepsis (OR=3.67), while Chan et al. similarly found an increased risk of sepsis (OR=1.56) in those undergoing elective lumbar surgery with a recent COVID-19 diagnosis [5,16].

Moreover, we observed an almost seven-fold increase in the risk of pneumonia, which has been commonly associated with COVID-19 patients undergoing elective orthopaedic surgery [16,17]. Similarly, Deng et al. found a significantly elevated risk of pneumonia (OR=6.46) in patients with a positive COVID-19 diagnosis within four weeks of elective surgery, noting that this increased risk could persist up to eight weeks before surgery [16]. These increases in various medical complication rates in the COVID-19 cohort may also further explain the significantly greater all-cause readmission rates this cohort exhibited.

As a result of the higher complication rates in this patient population, hospital stays are significantly prolonged (7.96 vs. 4.46 days), and 30-day costs increase substantially ($9,464 vs. $7,543), driven by the need for more intensive care and extended recovery periods. These extended hospitalizations and increased costs impose a significant burden on healthcare systems, leading to greater resource utilization and potentially straining the availability of critical care services.

Although no professional spine societies have established specific guidelines for the timing of elective spine surgery in patients with recent COVID-19, recommendations from other medical organizations can provide valuable guidance. In a joint collaboration, the American Society of Anesthesiologists (ASA) and Anesthesia Patient Safety Foundation (APSF) advise against elective surgeries within two weeks of a COVID-19 infection, recommending a delay of two to seven weeks for those with mild disease, and beyond seven weeks for patients with ongoing symptoms [18]. Additionally, a systematic review of 14 studies and professional guidelines suggested that prior to elective total joint arthroplasty, patients with asymptomatic COVID-19 should be rescheduled four to eight weeks after testing positive, and those with severe or critical illness should delay elective surgery for at least 12 weeks after hospital discharge [4]. These recommendations, alongside our study’s findings, support the consideration of at least a two-week delay before proceeding with elective lumbar spine surgery.

This study has several limitations. The main limitation is the lack of granular data in the PearlDiver database, which only allowed us to identify patients with a positive COVID-19 diagnosis, without the ability to stratify based on infection severity. Given the routine use of pre-admission COVID-19 testing and a unifying ICD-10 code for positive COVID-19 diagnosis, it is possible that some patients who tested positive were asymptomatic, as 32.4% of documented COVID-19 cases are asymptomatic [19]. We were unable to differentiate between symptomatic and asymptomatic cases or assess vaccination status, which could have impacted disease severity. As a result, sub-analysis by disease severity was precluded, potentially introducing heterogeneity in the COVID-19 cohort. As some patients are asymptomatic, patients in our control cohort may have also had an undocumented COVID-19 infection. However, due to routine preoperative COVID-19 screening during the pandemic, this potential confounder may be partially mitigated. Further, there may be residual confounding due to variables not assessed in the present study, such as stratification by lumbar fusion approach, alluded to by the large discrepancy in hospital readmission rates. This large discrepancy in hospital readmission rates may also be partially attributed to the nature of administrative claims databases due to an inherent lack of granularity, since it is capable of checking the instance of a new inpatient record, but cannot accurately verify that the readmission was related to postoperative complications related to the spinal fusion surgery. However, rigorous statistical methodology through propensity score matching and robust multivariable regression was employed to mitigate such confounding. Lastly, we could not account for variations in viral strains over the study period, which may have differing complication profiles.

## Conclusions

Patients with a positive COVID-19 diagnosis within two weeks of elective lumbar spine fusion are at a significantly higher risk of medical complications and readmissions. While this study cannot definitively recommend whether to proceed with surgery in these patients, we advise spine surgeons to carefully assess infection severity and comorbidities, and consider a delay of at least two weeks for non-urgent spine cases. As stratification by disease severity and vaccine status was precluded from this analysis, individualized risk stratification remains essential, and further institutional studies are needed to develop definitive guidelines.
